# Spatial distribution of 6*s*^2^ lone electron pair in Pb_4_Na(PO_4_)_3_ and stereochemical activity of the 6*s*^2^ electron cloud in lead-bearing apatites

**DOI:** 10.1107/S2052520625010844

**Published:** 2026-02-01

**Authors:** Kotomi Hirano, Hiroki Okudera

**Affiliations:** a Division of Geosciences and Civil Engineering, Graduate School of Natural Science and Technology Kanazawa University Kakuma-machi Kanazawa Ishikawa920-1192 Japan; b School of Geosciences and Civil Engineering, College of Science and Engineering Kanazawa University Kakuma-machi Kanazawa Ishikawa920-1192 Japan; University of Geneva, Switzerland

**Keywords:** Pb_4_Na(PO_4_)_3_, lone electron pair, electron density, anion channel

## Abstract

Single crystals of lacunary apatite structure-type compound Pb_4_Na(PO_4_)_3_ were prepared with a flux/solid-state-reaction technique and consecutive melt-growth to examine spatial distribution and stereochemical activity of the 6*s*^2^ lone electron pair of Pb^2+^ using X-ray diffraction. The target compound crystallized in space group *P*6_3_/*m* with unit-cell edge lengths *a* = 9.7345 (12) Å and *c* = 7.2130 (18) Å without any indication of configurational ordering of Pb^2+^ and Na^+^ at the crystallographic *A*1 site. The anion channel running through the structure was confirmed as vacant. Residual density attributable to the deformed 6*s*^2^ electron cloud was found 0.6 Å apart from the *A*2-site position on the *A*2 triangle normal to **c**. The lack of a channel-site anion induced shrinkage of the unit cell and the O3 trigonal antiprism in the *xy* plane, and an increase in the twist angle of the *A*1O_6_ trigonal metaprism compensated for the shrinkage of the latter to keep the volume of the *A*1O_9_ coordination polyhedron constant. Systematic comparison of the size of the *A*2 triangle in Pb_4_Na(PO_4_)_3_ and other apatite-type compounds indicates no contribution from the 6*s*^2^ electron orbital on the size of the *A*2 triangle and limited space-filling ability of the orbital. *A*2 cations are attached on the periphery of a large void formed by the in-plane distortion of the hexagonal close packed arrangement of (*B*O_4_)^3−^ complex anions. In other words, a face-sharing array of *A*2 octahedra was inserted ‘as a tube’ through the framework. The sizes of *A*1O_9_ and *B*O_4_ polyhedra and their framework define the maximum size of the *A*2 triangle, while the triangle can easily be shrunk by attraction from an anion, such as F^−^, in the channel.

## Abstract 2

In lacunary apatite structure-type Pb_4_Na(PO_4_)_3_, residual density attributable to the deformed 6*s*^2^ electron cloud is found in the vicinity of *A*2-site Pb^2+^ on regular *A*2 triangle normal to **c**. Systematic comparison on the size of the *A*2 triangle with other apatite-type compounds indicates limited stereochemical activity of the orbital.

## 1. Introduction

**Figure 1:**
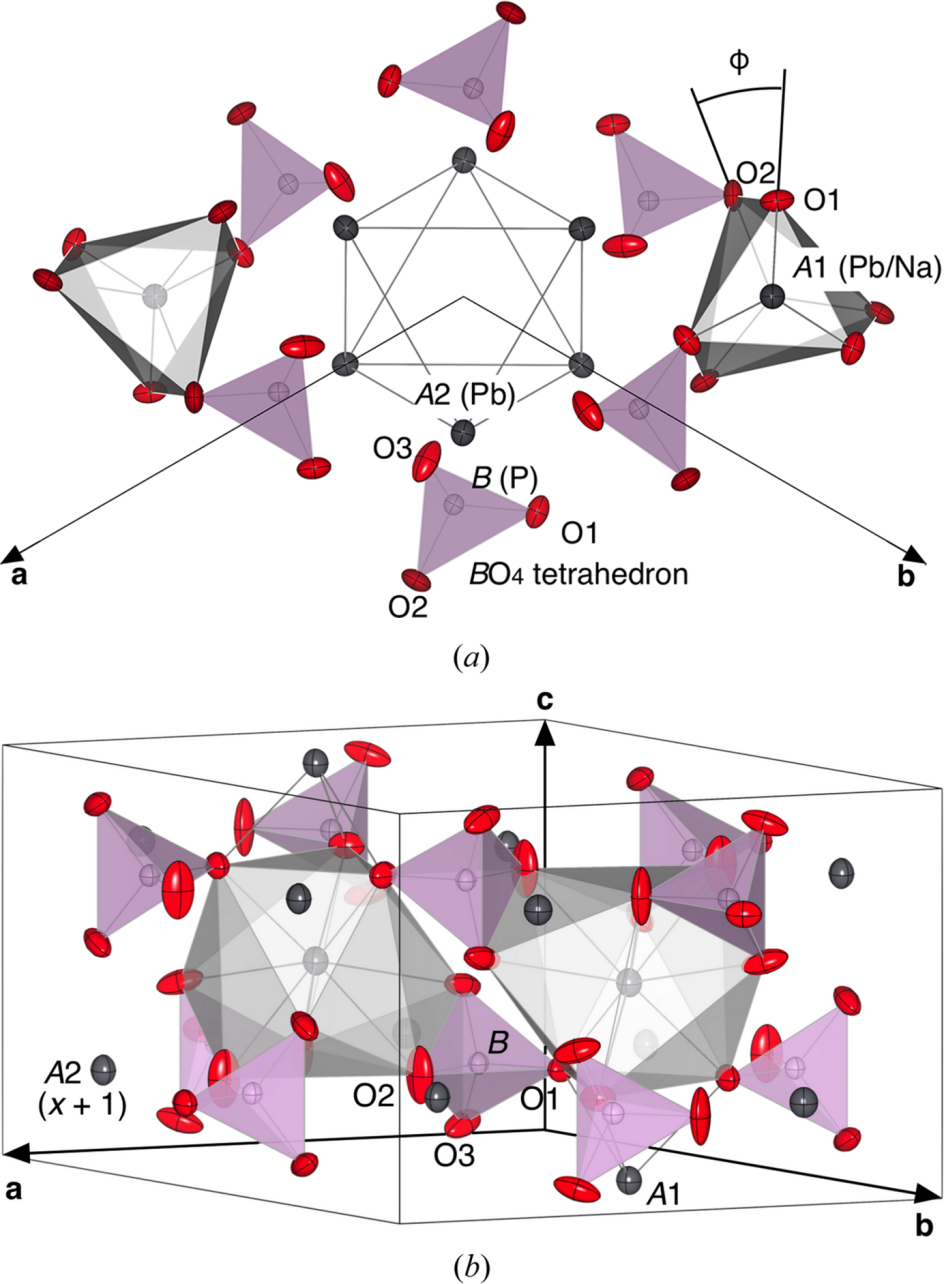
Structure of Pb_4_Na(PO_4_)_3_. (*a*) Projection in [001] with atoms in the range −0.6 < *x* < 0.6, −0.6 < *y* < 0.6, 0 < *z* < 1. Symmetry codes on atomic sites are abridged. *B*O_4_ polyhedra are drawn in purple. *A*1O_6_ trigonal metaprisms are drawn in grey with the definition of angle φ indicated. Edges of the *A*2 octahedron are drawn with grey lines. (*b*) Orthogonal projection of the structure with atoms in the range −0.2 < *x* < 1.2, −0.2 < *y* < 1.2, 0 < *z* < 1. *A*1O_9_ and *B*O_4_ polyhedra are drawn in grey and purple, respectively, to show their edge-sharing manner. All displacement ellipsoids at the 70% probability level. Drawn with *VESTA* (Momma & Izumi, 2011 ▸) software.

After the first reports of the structure of Ca_5_(PO_4_)_3_F by Mehmel (1930 ▸) and Náray-Szabó (1930 ▸), there are numerous articles to date regarding the structure of apatite-type compounds (hereafter referred to as apatites) for their industrial applications, such as an oxide ion conductor for solid oxide fuel cells operated under moderate temperature (Nakayama *et al.*, 1999 ▸; Okudera *et al.*, 2005 ▸; Ali *et al.*, 2009 ▸; Matsuura & Okudera, 2022 ▸). Their crystal chemistry, crystal structures with symmetry lowering (hettotypes), and their descriptions have been summarized in reviews (White & ZhiLi, 2003 ▸; Mercier *et al.*, 2005 ▸; Pasero *et al.*, 2010 ▸), and only a short description of the aristotype structure (space group *P*6_3_/*m*) is given below. The generalized formula of apatites is written as [*A*1_2_][*A*2_3_](*B*O_4_)_3_*X* (*Z* = 2), here *A*1, *A*2, *B* and *X* are designated also as atomic sites. The aristotype of their host structure can be depicted as a hexagonal close packed (hcp) array of (*B*O_4_)^3−^ complex anions with large in-plane distortion. This in-plane distortion forms four smaller and two larger octahedral voids (per unit cell). The smaller ones are arrayed parallel to **c** and filled by *A*1 cations at *z* ≃ 0 and 1/2 (Wyckoff position 4*f*). This site is coordinated by O1- and O2-site oxide anions to form an *A*1O_6_ trigonal prism with a metaprismatic twist angle φ and three more distant O3-site oxide anions which cap each of the square faces (Fig. 1 ▸). White & ZhiLi (2003 ▸) and Dong & White (2004 ▸) pointed out that the *A*1O_6_ metaprism is a key structural unit along with the *B*O_4_ unit, and the twist angle φ is a useful measure to validate the structure. The larger ones are large enough to accommodate three *A*2 cations around **c** at *z* = 1/4 and 3/4. A regular triangle made of these three *A*2 cations will be referred to as an *A*2 triangle. Six *A*2 cations in adjacent nets form a nearly regular trigonal antiprism with *A*2 cations on its apices (*A*2 octahedron). The face-sharing array of this octahedron along **c** forms an anion channel with the *X* anion as a guest at (0, 0, *z*) [*z* = 0 (Wyckoff position 2*b* with symmetry 3..), 1/4 (Wyckoff position 2*a* with symmetry 6..) or somewhere in between (Wyckoff position 4*e* with symmetry 3.. with a half occupancy)]. Six O3-site oxide anions on the periphery of this large void also form a highly oblate trigonal antiprism by two staggered O3 regular triangles (O3 triangle) normal to **c** in the middle of each *A*2 octahedron. Repulsion from O3-site oxide anions affects displacements and even the position of the *X* anion (Okudera, 2013 ▸; Matsuura & Okudera, 2022 ▸). Mercier *et al.* (2005 ▸) reported that unit-cell edge length *a* is governed by constraints inherent to the (*A*1O_6_)–(*B*O_4_) polyhedral arrangement, however, there was no in-detail discussion on repulsive forces inside the channel such as repulsion between O3 and *X* anions and direct interference of 6*s*^2^ lone electron pairs at the *A*2-site Pb^2+^ in lead-bearing apatites.

Contribution from the stereochemically active 6*s*^2^ lone electron pair on the stability of lacunary apatite structures has been mentioned by several authors (*e.g.* Mathew *et al.*, 1980 ▸; Krivovichev *et al.*, 2004 ▸; Kampf & Housley, 2011 ▸). They suggested the role of the space-filling 6*s*^2^ orbital as a substitute for the *X* anion to prevent the structure from collapse. Hirano & Okudera (2025 ▸) solved the structure of Pb_10_(PO_4_)_6_O and found commensurate modulation in sizes of *A*2 triangles in the manner ‘large-middle-small-middle(-large)’, which caused a 2 × **c** superstructure. Investigation of the structure showed significant local shrinkage of the channel due to attraction on *A*2-site Pb^2+^ from *X*-site O^2−^ in the channel and a simultaneous shift of O^2−^ out from the centre of the *A*2 triangle. The latter was attributed to the 6*s*^2^ electron cloud that thrusted O^2−^ out from its ideal position on the triangle plane. However, this structure also left some issues to be considered. First, the smallest *A*2 triangle [edge length *d*(*A*2*c*–*A*2*c*) = 3.871 (3) Å] was highly shrunk, suggesting that repulsive force among 6*s*^2^ electron clouds was rather weak. Second, the size of the largest *A*2 triangle [*d*(*A*2*a*–*A*2*a*) = 4.359 (3) Å] at the vacant part of the channel was comparable to that in Pb_5_(PO_4_)_3_Cl in which Cl^−^ and presumably space-filling 6*s*^2^ orbitals coexisted. Indeed, estimated bond valence sums (BVSs) for Cl^−^ in lead chlorapatites commonly exceeded its formal valence, in other words, the channel was tight even for Cl^−^ alone (Okudera, 2013 ▸). These observations might indicate rather limited ability of the 6*s*^2^ orbital to fill the space and also the highly subordinate nature of the size of the *A*2 octahedron.

Location, or deformation, of the 6*s*^2^ electron cloud and systematic variation in the sizes of the *A*2 triangle with- and without such a lone electron pair seem to be the keys to solve these questions. In spite of the long research history of apatite-type compounds, no accurate X-ray investigation of the 6*s*^2^ electron density had been reported to date. It is not surprising since Pb is a heavy X-ray absorber, and the presence of the *X* anion in the channel further hinders the precise investigation of electron density distribution inside the channel. Here, we report electron density distribution inside the vacant channel in one of the lacunary apatites, Pb_4_Na(PO_4_)_3_, after structure refinement with single-crystal X-ray diffraction. Stereochemical activity of the 6*s*^2^ lone electron pair at the *A*2 site will be briefly discussed based on relationships found among unit-cell edge length *a*, species of the *X* anion, and sizes of *A*2 and O3 triangles in the subjected compound and selected apatite-type compounds hitherto reported.

## 2. Experimental

### 2.1. Sample preparation

Pb_4_Na(PO_4_)_3_ (hereafter referred to as PNP) single crystals were prepared in two steps. A pre-grown powder of PNP was prepared by a flux/solid-state-reaction method. A mixture of PbO (99.5%; FUJIFILM Wako Pure Chemical Co., Japan), Bi_2_O_3_ (98.0%; Kanto Chemical Co., Inc., Japan), and NH_4_H_2_PO_4_ (99.0%; FUJIFILM Wako) reagents were mixed to attain Pb, Bi and P in a 9:1:6 molar ratio. Bi source was mixed in the starting material to check if we could incorporate Bi^3+^ in the apatite structure. Na_2_B_4_O_7_·10H_2_O (99.0%; FUJIFILM Wako) was added at 10 wt% of the mixture as a flux and also a Na source; the molar ratio of Pb and Na in the starting material was 4:0.74. The mixture was ground, charged into a porcelain crucible and heated in air. The temperature was raised from room temperature to 850°C over 1 h, held at this temperature for 24 h and slowly lowered to 600°C over 24 h. After holding the temperature for 2 h, the furnace was cooled down by turning the power off. This pre-grown powdery specimen was washed with water to remove the soluble component. All powder X-ray diffraction peaks from the specimen could be successfully indexed with a hexagonal apatite cell and a trace amount of cubic Pb_3_Bi(PO_4_)_3_ (Sahoo & Guru Row, 2010 ▸). A single-crystal specimen was grown from the melt of this pre-grown powdery specimen: the yielded powder was lightly ground, charged into a Pt capsule and heated in air. The temperature was raised from room temperature to 1100°C over 1 h and held at this temperature for 10 h. Then, the temperature was slowly lowered to 500°C over 120 h before turning the power off. Transparent crystals in euhedral shape (hexagonal prism with a hexagonal cap at one end) associated with a tiny amount of translucent white fragments were obtained. Weissenberg photographs of the transparent crystals indicated an aristotype apatite lattice of the crystal. The white fragments were confirmed as cubic Pb_3_Bi(PO_4_)_3_ with powder X-ray diffractometry.

In contrast to a recent report of Pb_7.4_Bi_0.3_Na_2.3_(PO_4_)_6_ (Hamdi *et al.*, 2007 ▸), preliminary qualitative- and quantitative-chemical analyses (a JEOL JSM-6010LV scanning electron microscope with an integrated energy dispersive spectrometer) showed no sign of bis­muth nor boron in transparent crystals from the batch. The averaged atomic ratio of Pb:Na:P at four points on two crystals was 3.90 (15):1.08 (3):3.03 (6) with reference to 12 oxygen atoms. Non-occurrence of Na-deficiency in the crystal could be explained as the formation of Pb_3_Bi(PO_4_)_3_ removed equal amounts of Pb and (PO_4_) from the system and caused overabundance of Na and (PO_4_) with respect to Pb in the system. Since the P:O ratio matched with that in the target composition and no Na-excess structure was possible for charge neutrality, we concluded at this stage that the single crystals from the batch had the target composition with no *X*-site anion nor point defect at cation sites.

### 2.2. Data collection and structure analysis

A single-crystal specimen was ground into a sphere of *d* = 195 µm. The intensities of Bragg reflections and values of θ were measured at room temperature on a Rigaku AFC-5S automated four-circle diffractometer with graphite-monochromatized Mo *Kα* radiation. The ω–2θ scan method with scan width 1.5° + 0.35tanθ and scan speed of 4° min^−1^ was employed at the data collection. 19672 (19658 for space group *P*6_3_/*m*) reflections in a full reciprocal sphere were measured up to 2θ = 90°. Space groups *P*31*c* and *P*3*c*1 were ruled out for violation of systematic absence of *hh*2*h**l* for odd *l* and *h**h*0*l* for odd *l*. Relationships found among Bragg positions and observed intensities suggested the Laue symmetry 6/*m*. There was no apparent scattering power at 000*l* with *l* ≠ 2*n* (*n*: integers) Bragg positions, which restricts the possible space group of the specimen to be *P*6_3_/*m* or *P*6_3_. Agreements among symmetry-equivalent reflections under 6/*m* (centrosymmetric) and 6 (noncentrosymmetric) point groups were virtually the same in spite of relatively large anomalous dispersion coefficients of Pb, and space group *P*6_3_/*m* was employed in the following examinations. Thus, ordering of Na at the *A*1 site was ruled-out at this stage. The least-squares fitting of the peak positions of 11 intense reflections in the range 43.9° < 2θ < 47.9° resulted in the unit-cell edge lengths of *a* = 9.7345 (12) Å and *c* = 7.2130 (18) Å after calibration with Si (Okada & Tokumaru, 1984 ▸). See Table 1 ▸ for crystallographic details and further experimental conditions.

The intensity data were converted to |*F*_obs_| and their standard uncertainties (s.u.s: σ), after applying Lorentz, polarization and spherical absorption corrections (*μr* = 5.56). Averages over equivalent reflections for the 6/*m* point group were taken, and the averages which obeyed conditions |*F*_obs_| ≥ 3σ(*F*_obs_) and |*F*_obs_|_max_ < 1.5|*F*_obs_|_min_ among equivalents were used in the structure refinements with weights of σ^−2^. The least-squares program *LSGCEX* (Kihara, 1990 ▸) was used for structure refinements with variables including one scale and one isotropic extinction factor [type I with the Lorentzian mosaic spread of Becker & Coppens (1974 ▸)]. Neutral form factors and a low-angle threshold for diffraction data (0.25 ≤ sinθ/λ) were employed after examinations noted in Okudera (2013 ▸). Neutral form factors for respective atoms and their anomalous dispersion terms were taken from *International Tables for Crystallography*, Vol. C.

**Figure 2:**
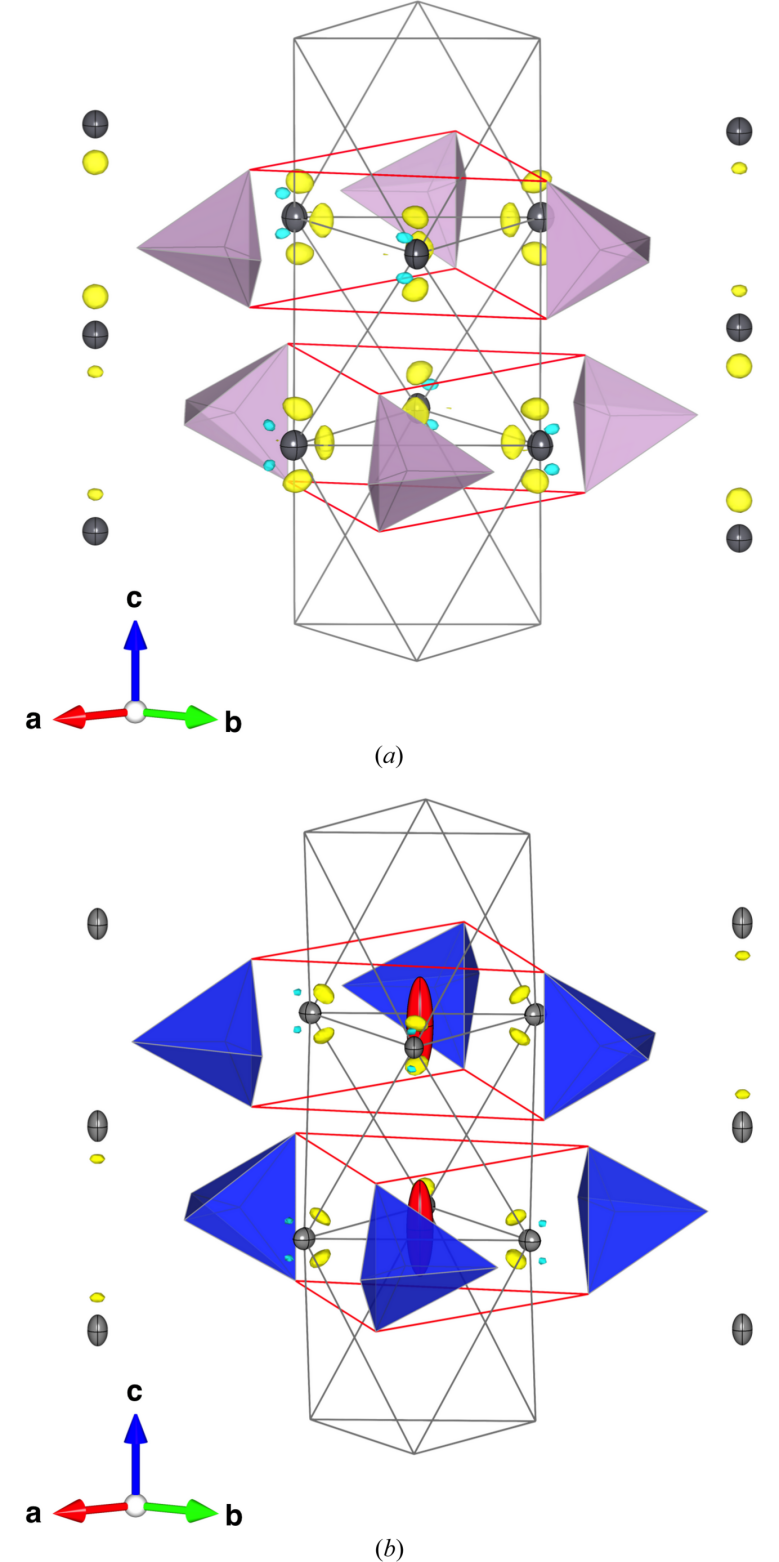
Residual density, Δρ, map with atoms in the range −0.5 < *x* < 0.5, −0.5 < *y* < 0.5, −0.1 < *z* < 1.1. *B* and O1–O3 atoms are abridged for clarity. O3 triangles and *A*2 octahedra are drawn in red and grey, respectively. All displacement ellipsoids are drawn at the 70% probability level. Purple and blue tetrahedra are (PO_4_)^3−^ and (SiO_4_)^4−^ complex anions, respectively. Isosurface: ± 1.5 e Å^−3^. Yellow: positive; pale blue: negative. (*a*) Pb_4_Na(PO_4_)_3_. (*b*) La_9.33_(SiO_4_)_6_O_2_ (Okudera *et al.*, 2005 ▸). *X*-site oxide anions are shown in red. Drawn with *VESTA* (Momma & Izumi, 2011 ▸).

The refinements started from the atomic coordinates and anisotropic displacement parameters (ADPs) given for Pb_5_(PO_4_)_3_Cl [OP-4 in Okudera (2013 ▸)] but no *X* site. In the following calculations the ratio of Pb:Na was fixed as 4:1 and all atomic sites were assumed fully occupied based on the results of quantitative chemical analyses. As a first attempt, the *A*1 site was equi-partitioned by Pb and Na and the *A*2 site was assumed solely occupied by Pb as reported by Koumiri *et al.* (2000 ▸), and coordinates of atomic sites with ADPs were refined. The calculation converged at *R*(*F*), *R*(*F*^2^) and *wR*(*F*) = 0.024, 0.040, 0.028 for 627 independent reflections with 38 parameters. Next, occupation of the *A*2 site by Na was allowed under above-mentioned constraints and restraints. The calculation converged at *R*(*F*), *R*(*F*^2^) and *wR*(*F*) = 0.017, 0.030, 0.019 with 39 parameters, indicating that a certain amount of Na was located at the *A*2 site as was also reported by Toumi & Mhiri (2008 ▸). Minimum and maximum Δρ were −1.80 e Å^−3^ at (0.97, 0.69, 0.80) and 2.64 e Å^−3^ at (0.33, 0.66, 0.58), respectively. No particular positive residual density attributable to an *X-*site anion was found on the *c* axis. The ADPs at the *A*2 site were refined also with third-rank tensors after Gram–Charlier expansion formalism (Johnson & Levy, 1974 ▸) for a highly skewed coordination environment at the site. Absolute values of some of third-rank tensors exceeded three times of their s.u.s. The largest among their absolute values was −3.4 (4) × 10^−5^ for *b*^222^ as expected from its coordination environment, namely, the absence of the *X* anion (Kuhs, 2003 ▸). Their absolute values, however, were small, and indeed no clear difference was found on Δρ maps after the refinements with and without those third-rank tensors. Details of structure refinement and refined structural parameters after the refinement with harmonic ADPs are given in Tables 1 ▸ and 2 ▸, respectively. Selected interatomic distances, bond angles, polyhedral volumes and bond-valence sums are given in Table 3 ▸. The residual density map after the refinement is shown in Fig. 2 ▸.

## 3. Results and discussion

### 3.1. Structure

The PNP specimen crystallized in an aristotype *P*6_3_/*m* apatite host structure with a vacant channel. In contrast to the previously reported structure, 11.4% of total Na was located at the *A*2 site in the present specimen instead of only 2.1% in Toumi & Mhiri (2008 ▸). Calculated BVSs for Pb^2+^ and Na^+^ at the *A*2 site were 1.99 and 0.86 after Brown & Altermatt (1985 ▸), 1.95 and 0.83 after Gagné & Hawthorne (2015 ▸), and 1.89 for Pb^2+^ after Krivovichev & Brown (2001 ▸), indicating that the refined coordinates represented the position of Pb, and an actual position of Na would be a little closer to the O2-site position to use up its charge. The mean-square displacements (MSDs), 〈*u*^2^〉 (Å^2^), at the *B* site were nearly isotropic and the smallest among all atomic sites as in most of the structures hitherto reported. Refined ADP values at O sites were larger and more anisotropic than those in natural pyromorphite [Pb_5_(PO_4_)_3_Cl], and those were closer to the values in natural vanadinite [Pb_5_(VO_4_)_3_Cl] (Okudera, 2013 ▸) and artificial La_9.33_(SiO_4_)_6_O_2_ (Okudera *et al.*, 2005 ▸). The libration mode of the (PO_4_)^3−^ complex anion in PNP was an intermediate of roto-oscillation around the *B*—O1 bond in the above-mentioned natural lead chlorapatites and cradle-like motion with *B*–O2 as the unique axis in La_9.33_(SiO_4_)_6_O_2_. Since no particular mode was dominant, the refined shape of the *B*O_4_ tetrahedron in PNP was fairly regular with a quadratic elongation index of 1.0014 and bond-angle variance of 5.73° despite its large libration amplitude. Displacement ellipsoids at *A* sites were slightly elongated (23% at *A*1 and 35% at *A*2) in [001], while their anisotropy was small. Virtually isotropic atomic displacements at the *A*1 site ascertained full occupation of the site by cations, otherwise the MSDs reflect local displacement of the *A*1-site cation (at 1/3, 2/3, *z*) toward one of its neighbouring *A*1-site positions (at 1/3, 2/3, *z* ± 1/2) when such a position is vacant as in the case of Nd_9.33_(SiO_4_)_6_O_2_ (Okudera *et al.*, 2004 ▸) and La_9.33_(SiO_4_)_6_O_2_.

### 3.2. Residual density

Residual density, Δρ, is shown in Fig. 2 ▸ with the isosurface of 1.5 e Å^−3^. The structure of La_9.33_(SiO_4_)_6_O_2_ (Okudera *et al.*, 2005 ▸) was re-refined for comparison purpose with a low-angle threshold 0.25 ≤ sinθ/λ and the same extinction formalism employed in the present study. The |*F*_obs_|_max_ < 1.1|*F*_obs_|_min_ threshold on structure refinement was kept unchanged. No notable difference was found on convergence of the least-squares cycles, refined structure and residual density from previously reported ones. Residual density after the calculation is also shown in Fig. 2 ▸.

There were some characteristic positive and negative residues in the vicinity of the *A*2 site position. Two accumulations of positive residue, separated on the *xz* plane for a mirror at *z* = 1/4, and two accumulations of negative residue, also separated from *z* = 1/4 but slightly out from the *xz* plane, were commonly found on PNP and La_9.33_(SiO_4_)_6_O_2_ despite absence of 6*s*^2^ electrons at *A*2-site La^3+^ in the latter. The appearance of positive residues on the triad axis which run through the *A*1 site could not be ascribed to 6*s*^2^ electrons at the site for the same reason. On the other hand, accumulation of positive residue at *z* = 1/4 in the vicinity of the *A*2 site was unique in PNP. Its maximum (2.61 e Å^−3^) was located at (−0.01, 0.19, 1/4), which was separated 0.6 Å from the *A*2 site position on the line to the centre of the *A*2 triangle, in contrast to speculations on an appearance of 6*s*^2^ electrons at the position opposite to the *A*2—O2 bond (*e.g*. Mathew *et al.*, 1980 ▸; Krivovichev *et al.*, 2004 ▸). Indeed, the appearance of this accumulation had a striking resemblance to electron localization function in the vicinity of *A*2-site Pb^2+^ in hexagonal Pb_5_(AsO_4_)_3_Cl after density functional theory calculations (Cametti *et al.*, 2022 ▸). The peak of observed accumulation was found on the opposite side with respect to the line, but spatial difference was small. So, the residue had attributes expected for the 6*s*^2^ electron cloud. More consideration, however, seemed necessary on its separation from the *A*2 site position. Similarly, prominent accumulation of positive residue was found at approximately 1.0 Å from the Bi core in Bi_2_WO_6_ (Okudera *et al.*, 2018 ▸) despite expectedly close attractions on electrons from respective atom cores. Stereochemical activity of 6*s*^2^ electrons will be nonetheless discussed in the following sections.

### 3.3. Unit-cell edge lengths and some characteristic values in PNP and chemically similar apatites

Some geometric parameter values in the PNP structure were compared first with those in closely related compounds, namely, normal and lacunary lead phosphate apatites (Table 4 ▸). Pb_5_(PO_4_)_3_Cl (Okudera, 2013 ▸) and Pb_5_(PO_4_)_3_Br (Liu *et al.*, 2011 ▸) in Table 4 ▸ have the *X* anion at *z* ≃ 0 with BVS values of 1.25 and 1.50, respectively. Unit-cell edge lengths *a* and *c* of the present PNP specimen were the smallest among these compounds. Variations in *c* showed that the presence or absence, and even the size, of the *X* anion in the channel had little effect on unit-cell edge length *c*. Differences in volume of the *A*1O_9_ polyhedron could be ascribed to differences in the sizes of Na^+^, Pb^2+^ and K^+^ at the *A*1 site in an increasing order (Shannon, 1976 ▸). A high population (25%) of point defects at the *A*1 site had little effect on the volume of the polyhedron in Pb_9_(PO_4_)_3_, and the unit-cell edge length *a* followed the increasing order of the sizes of *A*1-site cations among these lacunary apatites.

In contrast to the volume of the *A*1O_9_ polyhedron, unit-cell edge length *a*, edge lengths of *A*2 and O3 triangles [*d*(*A*2–*A*2) and *d*(O3–O3)] in PNP and Pb_9_(PO_4_)_6_ (Hata *et al.*, 1980 ▸) are virtually the same. These two edge lengths are a little larger in Pb_9_K(PO_4_)_6_, while differences were still small. *d*(*A*2–*A*2) in lacunary lead phosphate apatites are close also to that in Pb_5_(PO_4_)_3_Cl. O3 triangles are apparently larger in Pb_5_(PO_4_)_3_Cl and Pb_5_(PO_4_)_3_Br, validating the repulsion of O3-site O^2−^ from the *X* anion at *z* ≃ 0. As twist angle φ of the *A*1O_6_ trigonal metaprism indicated, these expansions are compensated by shift/rotation of the *B*O_4_ unit and a cooperative untwist of the metaprism. Size relationships in *a* and *d*(O3–O3) are concordant with that in *a* and the radius of the *X* anions (*e.g.* Sudarsanan & Young, 1974 ▸). These two compounds, however, showed a contrast in expansion of the *A*2 triangle: increase in *d*(*A*2–*A*2) was 1% in Pb_5_(PO_4_)_3_Cl and 5% in Pb_5_(PO_4_)_3_Br with reference Pb_9_(PO_4_)_6_. Intuitive understanding is that Br^−^ is too large to settle in the *A*2 octahedron of the Pb_5_(PO_4_)_3_ host structure without expanding the channel whereas Cl^−^ fits in the *A*2 octahedron. However, the *A*2 octahedron in Pb_5_(PO_4_)_3_Cl is still small for Cl^−^ as its BVS value indicated. The *A*2 octahedron can be larger also in Pb_5_(PO_4_)_3_Cl to realize the ideal coordination environment for Cl^−^, and a larger *A*2 octahedron would also be beneficial for 6*s*^2^ electrons. However, Cl^−^ is encapsulated in a small cavity in the Pb_5_(PO_4_)_3_Cl structure. Therefore, 6*s*^2^ electrons of Pb^2+^ at the *A*2 site should not be so active as to strongly affect the geometry of the host structure. Space-filling ability of those electrons would also be rather limited.

### 3.4. Stereochemical activity of 6*s*^2^ electrons at *A*2-site Pb^2+^

The above consideration on *d*(*A*2–*A*2) supports the idea that the size of the *A*2 triangle is primarily a subordinate of the sizes of *A*1O_9_ and *B*O_4_ polyhedra: the apatitic host structure is made of an hcp array of *B*O_4_ units with in-plane distortion primarily for the accommodation of the *A*1 cation in the smaller void, and face-sharing array of the *A*2 octahedron is inserted as a tube in resultant larger voids. To validate the idea, the sizes of *A*2 triangles were compared among chlor- and fluorapatites. Variation in size of the *A*1O_9_ polyhedron in mixed-cation cases will be reflected in unit-cell edge length *a*. The relationship between *a* and *d*(*A*2–*A*2) in lead apatites will differ from those in other apatites when interference from 6*s*^2^ electrons affects the structure.

**Figure 3:**
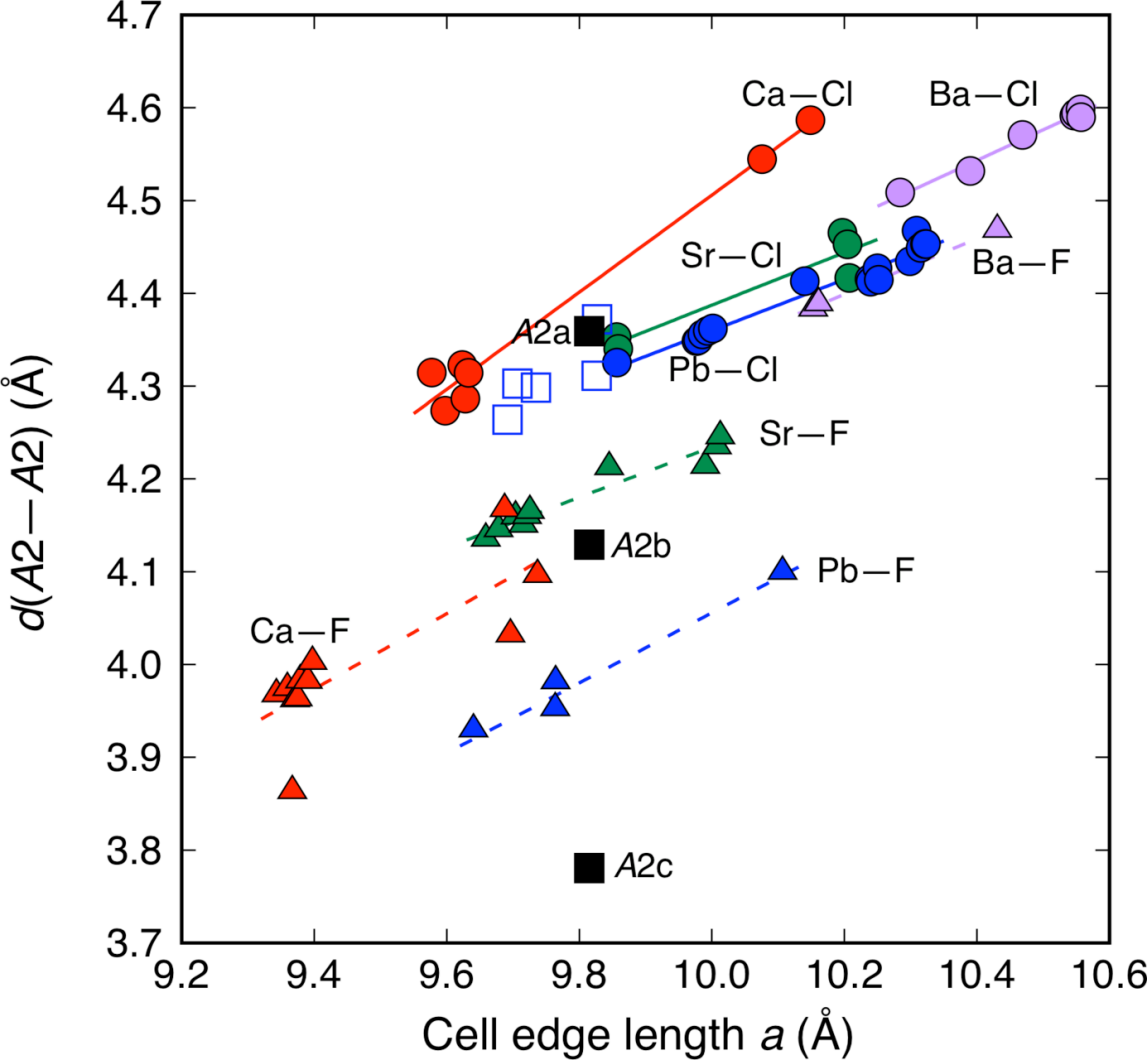
Variations in edge length of the *A*2 triangle with unit-cell edge length *a* in fluor-, chlor- and lacunary apatites together with those in Pb_10_(PO_4_)_6_O. Solid circles with solid lines: chlorapatites; solid triangles with dashed lines: fluorapatites; blue open squares: lacunarly apatites; black solid squares: three *A*2 triangles with different sizes in Pb_10_(PO_4_)_6_O. Purple: *A*2 = Ba; blue: *A*2 = Pb; green: *A*2 = Sr; red: *A*2 = Ca. Solid and dashed lines are drawn for guides for the eye after a least-squares fit for each combination of *A*2 and *X* ions.

Chlor- and fluorapatites (*A*2 = Ca, Pb, Sr and Ba, *B* = P, Mn, As and V) reported from 1971 onward were employed together with lacunary apatites and the 2 × **c** superstructure of Pb_10_(PO_4_)_3_O (Hirano & Okudera, 2025 ▸) for the following comparison. The 2 × **c** superstructure of Pb_10_(PO_4_)_3_O reported by Krivovichev & Engel (2023 ▸) was not used here for the reasons described in our previous study. When structures of solid-solution series were reported, only structures of end members were used for comparison. Up to 13% of heteroatom was allowed at the *A*2 site. Structures with multiple *X* sites are not used in this discussion. Unit-cell edge lengths, *d*(*A*2–*A*2) and the position of the *X* site in selected structures are listed in Table 5 ▸, together with the position and the BVS for the *X* anion after Brown & Altermatt (1985 ▸) for Ba^2+^—F^−^ and Ca^2+^—F^−^, and Brese & O’Keeffe (1991 ▸) for the other combinations. Variations in *d*(*A*2–*A*2) with unit-cell edge length *a* are shown in Fig. 3 ▸ with guide for the eye for each combination of *A*2 and *X* ions. As can be seen in Fig. 3 ▸, reported dependences of unit-cell edge length *a* on sizes of the *A*1 cation (*e.g.* Badraoui *et al.*, 2006 ▸), the *B* cation (*e.g.* Reinen *et al.*, 1986 ▸; Flis *et al.*, 2010 ▸) and the *X* anion (*e.g.* Sudarsanan & Young, 1974 ▸; Piotrowski *et al.*, 2002 ▸) held among compounds listed in Tables 4 ▸ and 5 ▸. BVS values for Cl^−^ exceeded its formal valence in all compounds and varied in the range 1.05∼1.40. Large *d*(*A*2–*A*2) values on calcium chlorapatites could be ascribed to the smaller size of Ca^2+^ under the inserted tube consideration. Despite large variations in BVS values, *d*(*A*2–*A*2) in all chlorapatites except *A*2 = Ca were found on single wide line. No characteristic feature was found on lead chlorapatites and the effect from the in-plane collision of 6*s*^2^ orbitals, if any, should be small. Sizes of *A*2 triangles in lacunary apatites including PNP were found on the extension of the line on the smaller *a* side as if the size of the triangle is a simple function of *a*. It is interesting to note that the largest one, *d*(*A*2*a*–*A*2*a*), in Pb_10_(PO_4_)_6_O also fell on the extension of this wide line: the size of the largest *A*2 triangle was not expanded as a counteraction for shrinkage of the other two triangles. Change in the size of the *A*2 triangle with a different *X* anion will be examined below by comparing its size in chlor- and fluorapatites.

The *d*(*A*2–*A*2) values in barium fluorapatites are also found on the wide line mentioned above. The unit-cell edge length *a* was reduced with change of *X* from Cl^−^ to F^−^ as it was expected from the reduction of the repulsion on O3-site O^2−^ from the *X* anion. Reduction in the *d*(*A*2–*A*2) values with the exchange was small but common among barium apatites and was approximately 0.12 Å. As positions of F^−^ with BVS values close to 1.0 indicated, F^−^ anions were located at their ideal positions in barium fluorapatites. This small but noticeable difference on *d*(*A*2–*A*2) indicated forced expansion of the Ba^2+^ octahedron due to repulsion from Cl^−^ and in fact how small it was. This difference was doubled in strontium apatites with BVS values for F^−^ in the range 0.9∼1.1 at *z* = 1/4. A gap between guides for the eye (Fig. 3 ▸) for barium and strontium fluorapatites indicated that the fluoride anion at the centre of the *A*2 triangle shrunk the triangle in the latter to make its coordination environment ideal as happened in the structures of Ca_5_(AsO_4_)_3_F and Ca_5_(VO_4_)_3_F (Baikie *et al.*, 2007 ▸) and that this effect was larger on the larger *d*(*A*2–*A*2) side. This simple interpretation, however, did not apply to lead fluorapatites. As the residual density map for the PNP structure suggested, the 6*s*^2^ electron cloud could be directional and located inside the *A*2 triangle (Fig. 2 ▸). The far smaller *d*(*A*2–*A*2) on lead fluorapatites than their strontium analogues with the out-of-plane position of the *X* anion indicated that attraction on Pb^2+^ from F^−^ shrunk the *A*2 triangle and that 6*s*^2^ electron clouds at *A*2-site Pb^2+^ repelled F^−^ from the centre of the triangle. The size of the smallest *A*2 triangle in the Pb_10_(PO_4_)_6_O structure indicated how much the Pb^2+^ triangle could shrink even with 6*s*^2^ electrons inside. However, the observed relationship between *a* and *d*(*A*2–*A*2) in lacunary apatites indicated that the triangle was not shrunk for lack of a large *X* anion. Simple and the same trends on lacunary apatites and chlorapatites indicated the subordinate nature on the size of the *A*2 octahedron to the (*A*1O_9_)—(*B*O_4_) framework which defines the maximum size of the *A*2 triangle. *A*2 cations stick on the periphery of the larger void as ‘decorations on the wall’ with reference to Krivovichev *et al.* (2004 ▸) in the distorted hexagonal net of (*B*O_4_)^3−^ complex anions, while they are easily pulled by the *X* anion for attraction.

In summary, the change in size of the *A*2 triangle was found primarily as a function of unit-cell edge length *a*. As BVS values for Cl^−^ indicated, the *A*2 octahedron was hard to expand by repulsion from the *X* anion, In other words, the anion channel was a face-sharing array of *A*2 octahedra inserted in the (*A*1O_9_)—(*B*O_4_) framework with geometric restraints, as suggested by Mercier *et al.* (2005 ▸). On the other hand, the triangle could be shrunk rather easily by attraction from the *X* anion. Repulsion among directional 6*s*^2^ electron orbitals might exist inside of the anion channel in lead-bearing apatites, but this repulsion had no effect on unit-cell edge length *a* nor the size of the *A*2 triangle. However, stereochemical activity of the *A*2-site 6*s*^2^ electrons was still potent enough to repel the *X* anion from the centre of the *A*2 triangle at *z* = 1/4. In this sense, directional *X*-anion conduction through the channel is hard to realize on lead apatites, irrespective of *A*1, *B* and *X* ions.

## Supplementary Material

Crystal structure: contains datablock(s) I. DOI: 10.1107/S2052520625010844/ra5162sup1.cif

Structure factors: contains datablock(s) Pb4Na(PO4)3. DOI: 10.1107/S2052520625010844/ra5162Isup2.hkl

CCDC reference: 2513045

## Figures and Tables

**Table 1:** Experimental details

Crystal data	
Chemical formula	Pb_4_Na(PO_4_)_3_
*M*_r_	1136.70
Crystal system, space group	Hexagonal, *P*6_3_/*m*
Temperature (K)	294
*a*, *c* (Å)	9.7345 (12), 7.2130 (18)
*V* (Å^3^)	591.93 (16)
*Z*	2
*D_x_* (g cm^−3^)	6.38
Radiation type	Mo *K*α
μ (mm^−1^)	57.316
Crystal shape	Sphere
Crystal radius (mm)	0.095

Data collection	
Diffractometer	Rigaku AFC5S
Data collection method	ω–2θ scan
Scan speed (° min^−1^)	4
Repeat scan	Up to 3 times until |*F*_obs_| > 10σ(*F*_obs_)
Absorption correction	Spherical
*T*_min_, *T*_max_	0.006, 0.009
No. of measured, independent and |*F*_obs_| ≥ 3σ(*F*_obs_) reflections	19658, 1729, 707
*R*_int_ for all reflections collected	0.101
(sin θ/λ)_max_ (Å^−1^)	0.995

Refinement	
*R*(*F*), *wR*(*F*), *S*	0.017, 0.019, 1.56
No. of reflections	627
*R*_int_ for used reflections	0.033
No. of parameters	39
No. of restraints	3
Δρ_max_, Δρ_min_ (e Å^−3^)	2.64, −1.80

Computer programs: Rigaku control software, *LSGCEX* (Kihara, 1990 ▸), *VESTA* (Momma & Izumi, 2011 ▸).

**Table 2:** Extinction factor, atomic coordinates, and anisotropic displacement parameters (Å^2^) *U*_22_ = *U*_11_ and *U*_12_ = 1/2*U*_11_ at the *A*1 site, *U*_13_ = *U*_23_ = 0 at *A*1, *A*2, *B*, O1 and O2 sites.

Extinction factor	0.012 (6)

Site and site symmetry	*A*1 and 4*f* 3..
Occupancy Pb	0.557 (2)
Occupancy Na	0.443
*x*	1/3
*y*	2/3
*z*	0.00877 (9)
*U*_11_	0.01344 (16)
*U*_33_	0.0166 (3)

Site and site symmetry	*A*2 and 6*h m*..
Occupancy Pb	0.962
Occupancy Na	0.038
*x*	0.00130 (3)
*y*	0.25558 (3)
*z*	1/4
*U*_11_	0.01270 (11)
*U*_22_	0.01554 (12)
*U*_33_	0.01904 (12)
*U*_12_	0.00706 (9)

Site and site symmetry	*B* and 6*h m*..
Occupancy P	1
*x*	0.3996 (2)
*y*	0.3785 (2)
*z*	1/4
*U*_11_	0.0099 (6)
*U*_22_	0.0088 (6)
*U*_33_	0.0100 (6)
*U*_12_	0.0049 (5)

Site and site symmetry	O1 and 6*h m*..
Occupancy O	1
*x*	0.3232 (7)
*y*	0.4827 (7)
*z*	1/4
*U*_11_	0.025 (3)
*U*_22_	0.013 (2)
*U*_33_	0.017 (2)
*U*_12_	0.013 (2)

Site and site symmetry	O2 and 6*h m*..
Occupancy O	1
*x*	0.5204 (8)
*y*	0.1019 (8)
*z*	1/4
*U*_11_	0.019 (3)
*U*_22_	0.019 (3)
*U*_33_	0.071 (6)
*U*_12_	0.015 (2)

Site and site symmetry	O3 and 12*i* 1
Occupancy O	1
*x*	0.3479 (7)
*y*	0.2685 (6)
*z*	0.0808 (6)
*U*_11_	0.044 (3)
*U*_22_	0.0221 (18)
*U*_33_	0.0151 (17)
*U*_12_	0.023 (2)
*U*_13_	−0.0100 (17)
*U*_23_	−0.0072 (14)

**Table 3:** Selected interatomic distances (Å), bond angles (°), polyhedral volume (Å^3^) and bond-valence sums Bond-valence parameters were taken from Brown & Altermatt (1985 ▸) (B&A), Krivovichev & Brown (2001 ▸) (K&B) and Gagné & Hawthorne (2015 ▸) (G&H).

*A*1 site	
*A*1–O1 (×3)	2.463 (6)
*A*1–O2 (×3)	2.717 (7)
*A*1–O3 (×3)	2.913 (7)
Mean value	2.696 (6)
BVS for Pb^2+^	2.09 (B&A), 2.16 (K&B), 2.16 (G&H)
BVS for Na^+^	0.91 (B&A), 0.91 (G&H)
	
*A*2 site	
*A*2–O1	2.790 (6)
*A*2–O2	2.262 (13)
*A*2–O3 (×2)	2.612 (5)
*A*2–O3 (×2)	2.529 (5)
Mean value	2.556 (6)
BVS for Pb^2+^	1.99 (B&A), 1.89 (K&B), 1.95 (G&H)
BVS for Na^+^	0.86 (B&A), 0.83 (G&H)
	
*B* site	
*B*–O1	1.528 (9)
*B*–O2	1.536 (10)
*B*–O3 (×2)	1.533 (5)
Mean value	1.533 (7)
BVS	5.03 (B&A)
O1–*B*–O2	111.2 (5)
O1–*B*–O3 (×2)	111.4 (3)
O2–*B*–O3 (×2)	108.5 (3)
O3–*B*–O3	105.4 (3)
Volume (Å^3^)	1.84 (1)

**Table 4:** Unit-cell edge lengths (Å), polyhedron *A*1O_9_ volume (Å^3^), *A*2 and O3 triangle edge lengths (Å), and twist angle φ (°) of the *A*1O_6_ trigonal metaprism in selected lead phosphate apatites

	*a*	*c*	*v*(*A*1O_9_)	*d*(*A*2–*A*2)	*d*(O3–O3)	φ	Reference
Pb_4_Na(PO_4_)_3_	9.7345 (12)	7.1230 (18)	37.45 (10)	4.2982 (7)	5.324 (9)	24.2 (4)	This study
Pb_9_(PO_4_)_6_	9.826 (4)	7.357 (3)	39.3 (3)	4.311 (3)	5.35 (3)	27.3 (12)	Hata *et al.* (1980 ▸)
Pb_4_K(PO_4_)_3_	9.827 (1)	7.304 (1)	40.19 (18)	4.372 (2)	5.402 (14)	28.8 (5)	Mathew *et al.* (1980 ▸)
Pb_5_(PO_4_)_3_Cl†	9.983 (1)	7.341 (1)	38.41 (13)	4.3525 (11)	5.643 (10)	18.0 (4)	Okudera (2013 ▸)
Pb_5_(PO_4_)_3_Br	10.0622 (5)	7.3575 (3)	39.70 (15)	4.5252 (16)	5.668 (14)	17.0 (4)	Liu *et al.* (2011 ▸)

† Averages of two natural specimens.

**Table 5:** Unit-cell edge lengths (Å), *A*2 triangle edge length (Å), *z*-coordinate of the *X* site and BVS for the *X* anion in hitherto reported chlorapatites, fluorapatites and selected related apatites *z*-coordinate of *X* site was taken at 0 ≤ *z* < 1/2. *z* < 1/4 when possible.

Composition	Method	*A*1	*a*	*c*	*d*(*A*2–*A*2)	*z* of *X*	BVS for *X*	Reference
Chlorapatites								
*A*2 = Ca								
Ca_4.78_Na_0.22_(PO_4_)_3_Cl_0.78_	Single	Ca	9.5773 (13)	6.8033 (6)	4.3149 (10)	0.0527 (6)	1.13	Matsuura & Okudera (2022 ▸)
Ca_5_(PO_4_)_3_Cl	Single	Ca	9.598 (2)	6.776 (4)	4.2736 (15)	0.0677 (4)	1.31	Hughes *et al.* (1989 ▸)
Ca_5_(PO_4_)_3_Cl_U†	Single	Ca	9.6233 (2)	6.7784 (3)	4.3229 (15)	0.060 (1)¶¶	1.18	Luo *et al.* (2009 ▸)
Ca_5_(PO_4_)_3_Cl	Single	Ca	9.628 (5)	6.764 (5)	4.2864 (19)	0.0562 (6)	1.23	Mackie *et al.* (1972 ▸) (averaged structure)
Ca_5_(PO_4_)_3_Cl_Th‡	Single	Ca	9.6330 (2)	6.7834 (2)	4.3229 (15)	0.059 (1)	1.20	Luo *et al.* (2009 ▸)
Ca_5_(AsO_4_)_3_Cl	Single	Ca	10.076 (1)	6.807 (1)	4.5446 (14)	0.1263 (5)	1.15	Wardojo & Hwu (1996 ▸)
Ca_5_(VO_4_)_3_Cl	Single	Ca	10.1490 (13)	6.7957 (18)	4.5865 (17)	0.1691 (6)	1.26	Matsuura & Okudera (2022 ▸)

*A*2 = Sr								
Sr_5_(PO_4_)_3_Cl_Th§	Single	Sr	9.8562 (3)	7.2095 (4)	4.3531 (8)	0	1.27	Luo *et al.* (2009 ▸)
Sr_5_(PO_4_)_3_Cl	Single	Sr	9.859 (1)	7.206 (2)	4.3401 (5)	0	1.26	Sudarsanan & Young (1974 ▸)
Sr_5_(AsO_4_)_3_Cl	Rietveld	Sr	10.1969 (1)	7.28108 (9)	4.466 (11)	0	1.05	Bell *et al.* (2009 ▸)
Sr_5_(VO_4_)_3_Cl	Single	Sr	10.2047 (11)	7.3040 (5)	4.4530 (10)	0	1.05	Matsuura & Okudera (unpublished)
Sr_5_(VO_4_)_3_Cl	Rietveld	Sr	10.2073 (1)	7.3067 (1)	4.417 (6)	0	1.07	Beck *et al.* (2006 ▸)

*A*2 = Pb								
Ca_2_Pb_3_(PO_4_)_3_Cl	Single	Ca	9.857 (1)	7.130 (2)	4.325 (2)	0	1.40	Kampf *et al.* (2006 ▸)
Pb_5_(PO_4_)_3_Cl	Single	Pb	9.977 (1)	7.351 (2)	4.3487 (18)	0	1.10	Dai & Hughes (1989 ▸)
Pb_5_(PO_4_)_3_Cl (OP-4)	Single	Pb	9.9791 (14)	7.3439 (11)	4.3493 (11)	0	1.25	Okudera (2013 ▸)
Pb_5_(PO_4_)_3_Cl (OP-1)	Single	Pb	9.9856 (10)	7.3318 (11)	4.3557 (10)	0	1.24	Okudera (2013 ▸)
Pb_5_(PO_4_)_3_Cl	Rietveld	Pb	9.9938 (1)	7.3397 (1)	4.3595 (9)	0	1.24	Flis *et al.* (2010 ▸)
Pb_5_(PO_4_)_3_Cl	Single	Pb	10.0017 (19)	7.3413 (16)	4.3620 (15)	0	1.23	Mills *et al.* (2012 ▸)
Ca_2_Pb_3_(AsO_4_)_3_Cl	Single	Ca	10.140 (3)	7.185 (4)	4.414 (5)	0	1.23	Rouse *et al.* (1984 ▸)
Pb_5_(AsO_4_)_3_Cl (OM-3)	Single	Pb	10.2382 (14)	7.4502 (12)	4.4154 (15)	0	1.10	Okudera (2013 ▸)
Pb_5_(AsO_4_)_3_Cl (OM-6)	Single	Pb	10.2396 (13)	7.4405 (24)	4.4358 (13)	0	1.11	Okudera (2013 ▸)
Pb_5_(VO_4_)_3_Cl	Single	Pb	10.250 (2)	7.454 (1)	4.426 (9)	0	1.08	Calos *et al.* (1990 ▸)
Pb_5_(AsO_4_)_3_Cl	Rietveld	Pb	10.2518 (2)	7.4482 (2)	4.4145 (11)	0	1.10	Flis *et al.* (2010 ▸)
Pb_5_(VO_4_)_3_Cl	Single	Pb	10.2990 (2)	7.3080 (1)	4.4347 (6)	0	1.14	Laufek *et al.* (2006 ▸)
Pb_5_(VO_4_)_3_Cl	Single	Pb	10.3090 (10)	7.3735 (17)	4.4675 (15)	0	1.06	Matsuura & Okudera (unpublished)
Pb_5_(VO_4_)_3_Cl	Single	Pb	10.315 (6)	7.337 (3)	4.3487 (18)	0	1.10	Dai & Hughes (1989 ▸)
Pb_5_(VO_4_)_3_Cl (OV-5)	Single	Pb	10.3217 (13)	7.3407 (13)	4.4530 (13)	0	1.10	Okudera (2013 ▸)
Pb_5_(VO_4_)_3_Cl (OV-1)	Single	Pb	10.3231 (9)	7.3399 (7)	4.4530 (13)	0	1.10	Okudera (2013 ▸)

*A*2 = Ba								
Ba_5_(PO_4_)_3_Cl	Single	Ba	10.284 (2)	7.651 (3)	4.509 (3)	0	1.39	Hata *et al.* (1979 ▸)
Sr_2_Ba_3_(AsO_4_)_3_Cl	Single	Sr	10.390 (1)	7.575 (1)	4.5318 (12)	0	1.39	Đordevic *et al.* (2008 ▸)
Ba_5_(MnO_4_)_3_Cl	Single	Ba	10.469 (1)	7.760 (1)	4.571 (3)	0	1.23	Reinen *et al.* (1986 ▸)
Ba_5_(VO_4_)_3_Cl	Rietveld	Ba	10.5468 (1)	7.7437 (1)	4.591 (5)	0	1.22	Beck *et al.* (2006 ▸)
Ba_5_(VO_4_)_3_Cl	Single	Ba	10.5492 (13)	7.7534 (8)	4.5942 (7)	0	1.20	Matsuura & Okudera (unpublished)
Ba_5_(VO_4_)_3_Cl	Single	Ba	10.5565 (1)	7.7584 (1)	4.5982 (9)	0	1.19	Roh & Hong (2005 ▸)
Ba_5_(AsO_4_)_3_Cl	Rietveld	Ba	10.5570 (1)	7.73912 (8)	4.590 (10)	0	1.21	Bell *et al.* (2008 ▸)

Fluorapatites								
*A*2 = Ca								
(Ca_0.879_Mn_0.121_)_5_(PO_4_)_3_F_0.74_	Single	Ca/Mn	9.343 (2)	6.8227 (10)	3.9677 (13)	0.25	0.89	Hughes *et al.* (1991 ▸)
(Ca_0.9582_Mn_0.0422_)_5_(PO_4_)_3_F_0.93_	Single	Ca/Mn	9.3596 (10)	6.8603 (10)	3.9745 (11)	0.25	0.88	Hughes *et al.* (1991 ▸)
Ca_5_(PO_4_)_3_F	Single	Ca	9.367 (1)	6.884 (1)	3.8634 (10)	0.25	1.05	Sudarsanan *et al.* (1972 ▸)
Ca_5_(PO_4_)_3_F_U¶	Single	Ca	9.3709 (2)	6.8849 (2)	3.9625 (12)	0.25	0.90	Luo *et al.* (2009 ▸)
Ca_5_(PO_4_)_3_F_Th††	Single	Ca	9.375 (2)	6.883 (3)	3.9632 (10)	0.25	0.90	Luo *et al.* (2009 ▸)
(Ca_0.9704_Sr_0.0296_)_5_(PO_4_)_3_F_0.89_	Single	Ca/Sr	9.379 (2)	6.8922 (7)	3.9827 (13)	0.25	0.87	Hughes *et al.* (1991 ▸)
(Ca_0.9372_Sr_0.0628_)_5_(PO_4_)_3_F	Single	Ca/Sr	9.390 (2)	6.9011 (8)	3.9822 (11)	0.25	0.87	Hughes *et al.* (1991 ▸)
Ca_5_(PO_4_)_3_F	Single	Ca	9.397 (3)	6.878 (4)	4.0025 (19)	0.25	0.84	Hughes *et al.* (1989 ▸)
Ca_5_(AsO_4_)_3_F	Rietveld	Ca	9.6873 (5)	6.9815 (3)	4.167 (3)§	0.245 (2)	0.83	Baikie *et al.* (2007 ▸)
Ca_5_(VO_4_)_3_F	Rietveld	Ca	9.6960 (3)	7.0170 (2)	4.03 (3)‡	0.252 (2)	0.80	Baikie *et al.* (2007 ▸)
Ca_5_(VO_4_)_3_F	Rietveld	Ca	9.7371 (1)	7.0063 (1)	4.096 (6)	0.25	0.73	Dong & White (2004 ▸)

*A*2 = Sr								
(Na,Ce)_2_Sr_3_(PO_4_)_3_F‡‡	Single	Na/Ce	9.659 (1)	7.182 (1)	4.1343 (12)	0.211 (1)‡	1.04	Rakovan & Hughes (2000 ▸)
Sr_5_(PO_4_)_3_F	Single	Sr	9.678 (3)	7.275 (5)	4.146 (3)	0.25	1.09	Swafford & Holt (2002 ▸)
Sr_5_(PO_4_)_3_F_Th§§	Single	Sr	9.7038 (4)	7.2723 (7)	4.1593 (8)	0.25	1.07	Luo *et al.* (2009 ▸)
(Sr_0.992_Nd_0.005_)_5_(PO_4_)_3_F	Single	Sr	9.7156 (4)	7.2810 (3)	4.1505 (15)	0.25	1.08	Corker *et al.* (1995 ▸)
Sr_5_(PO_4_)_3_F	Rietveld	Sr	9.7211 (2)	7.2869 (1)	4.160 (2)	0.2486 (3)	1.07	Aissa *et al.* (2004 ▸)
PbSr_9_(PO_4_)_3_F_2_	Rietveld	Sr	9.7268 (3)	7.2871 (1)	4.167 (3)	0.226 (3)	1.04	Badraoui *et al.* (2006 ▸)
Sr_5_(PO_4_)_3_F	Single	Sr	9.845 (7)	7.383 (4)	4.211 (5)	0.25	0.98	Pekov *et al.* (2010 ▸)
Sr_5_(AsO_4_)_3_F	Single	Sr	9.990 (1)	7.395 (1)	4.2139 (11)	0.25	0.98	Đordevic *et al.* (2008 ▸)
(Sr_0.982_Nd_0.012_)_5_(VO_4_)_3_F	Single	Sr	10.0077 (6)	7.434 (4)	4.2349 (15)	0.25	0.95	Corker *et al.* (1995 ▸)
Sr_5_(VO_4_)_3_F	Rietveld	Sr	10.01267 (7)	7.43169 (4)	4.246 (3)	0.220 (3)	0.91	Oka *et al.* (2022 ▸)

*A*2 = Pb								
Ca_2_Pb_3_(PO_4_)_3_F	Single	Ca	9.6402 (12)	7.0121 (8)	3.9298 (8)	0	0.62	Kampf & Housley (2011 ▸)
Pb_5_(PO_4_)_3_F	Single	Pb	9.7638 (6)	7.2866 (4)	3.9526 (19)	0.039 (4)	0.59	Fleet *et al.* (2010 ▸)
Pb_9_Sr(PO_4_)_6_F_2_	Rietveld	Pb	9.7662 (4)	7.2929 (2)	3.987 (6)	0.051 (3)	0.60	Badraoui *et al.* (2006 ▸)
Pb_3_(VO_4_)_3_F	Rietveld	Pb	10.10647 (15)	7.35582 (8)	4.1000 (3)	0.052 (3)	0.90	Oka *et al.* (2022 ▸)

*A*2 = Ba								
Ba_5_(PO_4_)_3_F	Single	Ba	10.153 (2)	7.733 (3)	4.383 (3)	0.219 (5)	1.16	Mathew *et al.* (1979 ▸)
Ba_5_(PO_4_)_3_F	Rietveld	Ba	10.1611 (2)	7.7322 (1)	4.389 (4)	0.2019 (7)	1.10	Aissa *et al.* (2004 ▸)
Ba_5_(VO_4_)_3_F	Rietveld	Ba	10.43080 (5)	7.86002 (4)	4.468 (3)	0.223 (3)	1.02	Oka *et al.* (2022 ▸)

Lacunary and superstructure apatites								
Pb_7.36_Bi_0.32_Na_2.08_Li_0.24_(PO_4_)_6_	Rietveld	Pb/Na	9.6916 (8)	7.1751 (7)	4.2639 (11)			Hamdi *et al.* (2007 ▸)
Pb_7.4_Bi_0.3_Na_2.3_(PO_4_)_6_	Rietveld	Pb/Na	9.7065 (7)	7.1705 (6)	4.3026 (9)			Hamdi *et al.* (2007 ▸)
Pb_10_(PO_4_)_6_O	Single	Pb	9.8151 (15)	14.8458 (11)	4.359 (3)			Hirano & Okudera (2025 ▸); *d*(*A*2*a*–*A*2*a*)
					4.128 (6)			Hirano & Okudera (2025 ▸); *d*(*A*2*b*–*A*2*b*)
					3.781 (3)			Hirano & Okudera (2025 ▸); *d*(*A*2*c*–*A*2*c*)

† (Ca_0.994_U_0.006_)_2_(Ca_0.993_U_0.007_)_3_(PO_4_)_3_Cl.

‡ (Ca_0.977_Th_0.023_)_2_(Ca_0.979_Th_0.021_)_3_(PO_4_)_3_Cl.

§ (Sr_0.985_Th_0.015_)_2_(Sr_0.990_Th_0.010_)_3_(PO_4_)_3_Cl.

¶ Ca_2_(Ca_0.986_U_0.014_)_3_(PO_4_)_3_F.

†† (Ca_0.999_)_2_(Ca_0.987_Th_0.013_)_3_(PO_4_)_3_F.

‡‡ (Na_2.12_Ce_1.18_La_0.64_Nd_0.34_)(Sr_5.74_Ba_0.22_)(P_6.06_Si_0.22_)O_24_(F_1.96_OH_0.02_Cl_0.02_).

§§ (Sr_0.993_Th_0.007_)_2_(Sr_0.993_Th_0.007_)_3_(PO_4_)_3_F.

¶¶ From CIF.

††† Averaged *d*(*A*2–*A*2) in *P*1 structure.
